# Prevalence and Factors Associated with Postpartum Depressive Symptoms Among Women Attending Postnatal Check-Ups at Two Hospitals in Yunnan Province, China: A Cross-Sectional Study

**DOI:** 10.3390/nursrep16070215

**Published:** 2026-06-25

**Authors:** Zhuoting Ying, Piyanut Xuto, Sujitra Chaiwuth

**Affiliations:** Faculty of Nursing, Chiang Mai University, 110/406 Intawaroros Rd., Suthep, Muang, Chiang Mai 50200, Thailand; zhuoting_ying@cmu.ac.th (Z.Y.); sujitra.c@cmu.ac.th (S.C.)

**Keywords:** doing-the-month, maternal mental health, nursing, perceived social support, postpartum depression, postpartum women, sleep quality

## Abstract

**Background**: Postpartum depression is a prevalent maternal mental health concern during the early postpartum period. Nevertheless, limited evidence is available regarding this condition among postpartum women in Yunnan Province, China. **Objectives**: To examine the prevalence and severity of postpartum depression and to identify factors associated with postpartum depression among postpartum women attending postnatal check-ups at two hospitals in Yunnan Province. **Methods**: A cross-sectional study was conducted with 168 postpartum women at 6 weeks postpartum at two hospitals in Yunnan Province. Participants were recruited by convenience sampling. Data were collected using the Edinburgh Postnatal Depression Scale, the Self-Rating Scale of Sleep, the Perceived Social Support Scale, and the Adherence to the “Doing-the-Month” Practice Scale. Descriptive statistics and binary logistic regression were used to analyze the data. **Results**: The prevalence of postpartum depressive symptoms was 44.6%. In the adjusted model, poor sleep quality was associated with higher odds of postpartum depressive symptoms (aOR = 5.95, 95% CI: 2.78–12.70), whereas high perceived social support was associated with lower odds of these symptoms (aOR = 0.33, 95% CI: 0.15–0.70). Adherence to doing-the-month practices was not significantly associated with postpartum depressive symptoms. **Conclusions**: Postpartum depressive symptoms were common at 6 weeks postpartum in this two-hospital sample from Yunnan Province. Routine postnatal care should incorporate early screening for depressive symptoms, the assessment of sleep quality, and strategies to strengthen perceived social support. These measures may facilitate the timely identification of women requiring further psychological evaluation and support.

## 1. Introduction

Postpartum depression (PPD) is a prevalent consequence of childbirth throughout the postpartum period [1]. The ICD-10 defines PPD as a mental and behavioral disorder occurring within 6 weeks after childbirth [2]. Globally, a meta-analysis indicates that the prevalence of PPD is approximately 12.0% in developed regions, compared with 15.0% in developing countries [3]. In China, the prevalence was reported to be 21.4%, exceeding the rates observed in several developed countries [3]. Notably, a recent study found that PPD affected 46.05% of women in Yunnan Province [4], which was considerably higher than the reported national prevalence of 21.4% in China [3].

PPD can have adverse consequences for both mothers and children. It may lead to persistent sadness, loss of interest, diminished capacity for pleasure, and decreased responsiveness to infant cues, potentially interfering with mother–infant bonding [5]. In addition, maternal PPD may be associated with delays in several domains of infant development during the first two years of life, including communication, social functioning, fine motor coordination, adaptive behavior, and gross motor development [6].

The biopsychosocial model proposed by Engel emphasizes that health and illness are shaped by the complex interactions among biological, psychological, and social factors [7]. Accordingly, factors associated with PPD can be broadly conceptualized within this framework as biological factors, such as mode of delivery, postpartum hemorrhage and obstetric complications; psychological factors, such as negative birth experiences, anxiety, and fear; and social factors, such as education, income, and marital status [8].

Sleep quality is an important consideration during the postpartum period, which is characterized by physiological and behavioral changes, physical discomfort, and increased demands related to newborn care [9]. Poor sleep quality may increase the risk of depression through several biological pathways, including dysregulation of the hypothalamic–pituitary–adrenal (HPA) axis, altered monoamine neurotransmission, increased inflammation and oxidative stress, and impaired neuroplasticity [10]. As a potentially modifiable factor, sleep quality may serve as an important target for the early identification and prevention of PPD. Perceived social support is also important for understanding PPD within the biopsychosocial framework. Evidence from previous studies indicates that greater perceived social support is associated with a lower likelihood of PPD. When women perceive adequate support from family, friends, and significant others, they are more likely to feel accepted, loved, and valued [11]. However, some mothers may experience loneliness and inadequate support from their partners and family members during the perinatal period, which may contribute to perinatal distress [12]. These findings underscore the importance of perceived social support as a clinically relevant psychosocial factor in the early identification and prevention of PPD. Doing-the-month is a culturally significant traditional Chinese postpartum practice in which women rest at home for about one month after childbirth and follow specific practices intended to restore bodily balance, including restrictions on physical activity, maintenance of body warmth, and dietary regulation [13]. These family-based practices may complement formal postpartum nursing care by providing rest, nutritional support, and assistance with household tasks and infant care. However, women may still require professional guidance and psychological support, and some restrictive practices should be re-evaluated in accordance with evidence-based postpartum care recommendations [14].

These three variables—sleep quality, perceived social support, and doing-the-month practices—were selected based on their theoretical relevance within the biopsychosocial framework and their clinical modifiability. Sleep quality during the postpartum period is influenced by infant-care demands and hormonal changes rather than pre-existing psychopathology and has been shown in prospective studies to precede the onset of depressive symptoms [15,16]. Perceived social support represents a subjective, clinically assessable protective factor supported by the stress-buffering model [17] and supported by substantial evidence from postpartum populations [11]. Doing-the-month practices were included as a culturally specific and locally relevant factor that has not been adequately studied in the multi-ethnic context of Yunnan, where the meaning and implementation of these practices may differ substantially from those in other Chinese populations. However, few studies have examined the combined associations of sleep quality, perceived social support, and doing-the-month practices with PPD within the complex ethnic, cultural, and geographic context of Yunnan Province. Women from different ethnic groups may differ in their preferences for antenatal care, facility-based delivery, and postpartum follow-up, indicating that maternal health service needs and expectations may vary across population groups in this multi-ethnic region of southwestern China [18]. Therefore, findings from other regions of China may not be directly generalizable to Yunnan. In addition, the association between doing-the-month practices and PPD remains unclear [19], particularly in ethnic minority areas, where research on postpartum confinement practices remains limited. Both the specific practices and women’s lived experiences of them may vary across settings and population groups [20]. Furthermore, a previous study conducted in Yunnan used an EPDS cut-off score of 12 [4]; however, validation evidence from mainland China indicates that a cut-off score of ≥10 may be more appropriate for Chinese women [21], suggesting that the selection of an appropriate EPDS cut-off score for women in Yunnan requires further consideration.

Thus, guided by the biopsychosocial model and informed by the existing literature, this study aimed to determine the prevalence and severity of postpartum depressive symptoms and to examine the associations between sleep quality, perceived social support, doing-the-month practices, and postpartum depressive symptoms among women in Yunnan Province, China. Consistent with the cross-sectional design, these variables were examined as clinically assessable risk indicators concurrently associated with postpartum depressive symptoms, rather than as established causal determinants. This distinction acknowledges the inherent limitation of cross-sectional designs in establishing temporality and causal relationships and is consistent with the standard methodological approach used in hospital-based postpartum mental health research [4,22].

## 2. Materials and Methods

### 2.1. Study Design

This study employed a cross-sectional design to assess the prevalence and severity of postpartum depressive symptoms and to examine the associations of sleep quality, perceived social support, and adherence to doing-the-month practices with postpartum depressive symptoms within a biopsychosocial framework. The study was reported in accordance with the Strengthening the Reporting of Observational Studies in Epidemiology (STROBE) guidelines.

### 2.2. Participants

This study recruited 177 women at 6 weeks postpartum from two hospitals in Yunnan Province, China: Kunming Angel Women & Children’s Hospital and Jingdong Yi Autonomous County People’s Hospital. The two hospitals were selected because they provide postpartum follow-up care and serve women from diverse areas of Yunnan Province, including urban, county-level, and multi-ethnic communities. Convenience sampling was used to recruit eligible participants who attended postpartum follow-up visits for routine 6-week postnatal check-ups during the data collection period. Participants were eligible if they met the following criteria: (1) were aged 20–39 years; (2) were 6 weeks postpartum; (3) had delivered a healthy, full-term infant; (4) were able to communicate, read, and write in Chinese; (5) provided informed consent and agreed to participate in the study; and (6) had access to a mobile phone and were able to use WeChat. The exclusion criteria were a history of severe psychiatric disorders, postpartum complications, and having an infant admitted to the neonatal intensive care unit (NICU) because of a critical health condition.

According to the events-per-variable (EPV) rule for logistic regression proposed by Peduzzi et al. [23], a minimum of 10 events per variable is generally recommended. The sample size was estimated using the following formula: sample size (*n*) = EPV × number of predictors/occurrence ratio. A previous study reported a PPD prevalence of 21.4% in China [3]. Therefore, based on an EPV of 10, an occurrence ratio of 0.214, and three factors, the minimum required sample size was 141. After accounting for an anticipated 20% rate of missing or invalid data, the target sample size was increased to 177 postpartum women.

### 2.3. Research Instrument

This study used five instruments to collect data. Permission to use each instrument was obtained before data collection. Cronbach’s alpha coefficients were calculated in a pilot test involving 15 postpartum women who met the same eligibility requirements as the participants in the main study.

#### 2.3.1. Demographic Data Form

The researcher developed a demographic questionnaire comprising items on maternal age, ethnicity, maternal education level, employment status, parity, and living with mother-in-law.

#### 2.3.2. Edinburgh Postpartum Depression Scale (EPDS)

The EPDS was originally developed by Cox et al. [24] and translated into Chinese by Lee et al. [25] to assess postpartum depressive symptoms during the postnatal period, including reduced enjoyment, self-blame, anxiety or worry, fear or panic, difficulty coping, sadness or misery, crying, sleep difficulty related to unhappiness, and thoughts of self-harm. The scale consists of 10 items rated on a 4-point Likert scale, with a total score ranging from 0 to 30. A cut-off score of 10 or higher was used to indicate possible postpartum depressive symptoms among Chinese women [21,25]. In this study, participants with EPDS scores of ≥10 were categorized as having postpartum depressive symptoms, whereas those with scores below 10 were classified as not having postpartum depressive symptoms. The EPDS demonstrated good internal consistency in this study, with a Cronbach’s alpha coefficient of 0.83.

#### 2.3.3. Self-Rating Scale of Sleep (SRSS)

The SRSS was developed in Chinese by Li et al. [26] to evaluate sleep quality. The scale assesses sleep quality over the previous month, including perceived sleep sufficiency, feeling rested after sleep, overall sleep quality, sleep duration, difficulty falling asleep, nighttime awakenings or early awakening, nightmares, use of sleep medication, daytime sleepiness, and the effect of sleep problems on daily functioning. The scale consists of 10 items rated on a 5-point Likert scale, with total scores ranging from 10 to 50, where higher scores indicate poorer sleep quality [26]. In this study, participants were divided into two groups according to the classification approach used by Jiang et al. [27]: participants with SRSS scores of >22 were classified as having low sleep quality, whereas those with scores of ≤22 were classified as having high sleep quality. This categorization was used to facilitate clinically interpretable comparisons between groups and the interpretation of odds ratios. The SRSS demonstrated good internal consistency in this study, with a Cronbach’s alpha coefficient of 0.85.

#### 2.3.4. Perceived Social Support Scale (PSSS)

The PSSS was developed by Zimet et al. [28] and translated into Chinese by Jiang [29] to assess perceived social support. The scale evaluates individuals’ subjective perceptions of the availability and adequacy of support from three sources: family, friends, and significant others. These sources reflect key interpersonal support factors, including emotional concern, practical help, encouragement, and a sense of being valued and supported. The PSSS consists of 12 items measuring support received. Each item is rated on a 7-point Likert scale, yielding a total score ranging from 12 to 84; higher scores indicate greater perceived social support [28]. In the absence of a universally validated cut-off score, the sample median was used to dichotomize the scores, thereby facilitating the interpretation of group comparisons and odds ratios. In this study, a sample median score of 66 was used as the cut-off value. Participants with scores of ≤66 were classified as having low perceived social support, whereas those with scores of >66 were classified as having high perceived social support. The PSSS demonstrated good internal consistency in this study, with a Cronbach’s alpha coefficient of 0.87.

#### 2.3.5. Adherence to the “Doing-the-Month” Practice (ADP) Scale

The ADP scale was developed in Chinese by Chien et al. [13] to assess adherence to doing-the-month practices. The scale covers common postpartum confinement behaviors, including rest and activity restriction, maintaining body warmth, dietary practices, personal hygiene-related restrictions, and other culturally prescribed postpartum health practices. The scale consists of 27 items rated on a 5-point Likert scale ranging from 0 to 4, yielding a total score ranging from 0 to 108 [13]. Because no established cut-off score was available, ADP scores were dichotomized at the sample median to facilitate group comparisons and the interpretation of odds ratios. In this study, the sample median score of 52 was used as the cut-off value. Participants with scores of ≤52 were classified as having low adherence, whereas those with scores of >52 were classified as having high adherence. The ADP scale demonstrated good internal consistency in this study, with a Cronbach’s alpha coefficient of 0.82.

### 2.4. Ethical Considerations and Data Collection

This study was approved by the Research Ethics Office, Faculty of Nursing, Chiang Mai University (Approval No. 133/2025-EXP079). Data collection was conducted between November 2025 and January 2026 at the two participating hospitals in Yunnan Province. After ethical approval and institutional permission had been obtained from both participating hospitals, eligible postpartum women were recruited during their scheduled 6-week postnatal check-ups. Potential participants were assessed for eligibility according to the inclusion and exclusion criteria after completing their routine medical examinations. The researcher explained the study objectives, procedures, confidentiality measures, and participants’ rights. Subsequently, a QR code linking to the electronic informed consent form and online questionnaire was provided. Electronic informed consent was required before participation. Women who selected “I consent to participate” completed the questionnaire independently on their mobile phones, with completion taking approximately 15–20 min. Those who did not provide consent were automatically directed out of the survey platform, and no data were collected. Participation was voluntary, and participants could skip sensitive questions or withdraw at any time without penalty or adverse effects on their clinical care. All responses were collected anonymously, maintained confidentially, and used solely for research purposes. The quality of the completed questionnaires was assessed before data analysis. Responses were considered invalid if the questionnaire was incomplete, was completed in considerably less than the expected minimum completion time of 15 min, or showed potentially careless response patterns, such as selecting the same response option throughout the questionnaire or providing highly repetitive responses. Of the 177 questionnaires collected, nine met one or more of these criteria and were excluded, leaving 168 valid questionnaires for the final analysis.

### 2.5. Data Analysis

Data analysis was performed using SPSS version 22.0, with statistical significance set at α = 0.05. Descriptive statistics, including frequencies and percentages, were used to describe participants’ demographic characteristics and key study variables. Univariable binary logistic regression was initially used to assess the crude association between each independent variable and postpartum depressive symptoms. Subsequently, multivariable binary logistic regression was used to examine the associations between sleep quality, perceived social support, adherence to doing-the-month practices, and postpartum depressive symptoms after adjustment for maternal age, ethnicity, educational level, employment status, parity, and living with a mother-in-law. Crude odds ratios, adjusted odds ratios, 95% confidence intervals, and *p*-values were reported.

Before the regression analyses were conducted and interpreted, the assumptions and diagnostic criteria for binary logistic regression were assessed, including the adequacy of the sample size, appropriate variable coding, multicollinearity, and model fit. Multicollinearity was evaluated using variance inflation factors, while model fit and performance were assessed using the Hosmer–Lemeshow goodness-of-fit test, Cox and Snell R^2^, Nagelkerke R^2^, and overall classification accuracy.

## 3. Results

### 3.1. Demographic Characteristics and Postpartum Depression Symptoms of the Participants

As shown in Table 1, 168 postpartum women attending postnatal check-ups at two hospitals in Yunnan Province participated in this study. Of these, 75 met the criteria for postpartum depressive symptoms, corresponding to a prevalence of 44.60% within the study sample. Most participants were aged ≥25 years (89.30%), identified as Han Chinese (72.60%), had attained a college or university education or higher (83.90%), and were employed (63.70%). The majority were primiparous (63.70%), and slightly more than half were living with their mother-in-law (51.20%). Overall, the sample consisted predominantly of adult, highly educated, employed, first-time mothers.

### 3.2. Descriptive Statistics of Study Variable

Table 2 indicates that the median postpartum depression score was 9 (IQR = 7). The median sleep quality score was 23 (IQR = 9). The median perceived social support score was 66 (IQR = 19). The median adherence to doing-the-month practices score was 52 (IQR = 36).

### 3.3. Univariable Logistic Regression Analysis of Postpartum Depressive Symptoms

As illustrated in Table 3, univariable logistic regression analyses identified several factors associated with postpartum depressive symptoms. Women with low sleep quality were significantly more likely to experience postpartum depressive symptoms compared with those with high sleep quality (OR = 6.71, 95% CI: 3.34–13.46, *p* < 0.001). In contrast, high perceived social support was significantly associated with lower odds of postpartum depressive symptoms (OR = 0.23, 95% CI: 0.12–0.44, *p* < 0.001). However, adherence to doing-the-month practices was not significantly associated with postpartum depressive symptoms. The univariable analysis of demographic factors is presented in the Supplementary Materials (see Supplementary Table S1).

### 3.4. Multivariable Logistic Regression Analysis of Factors Associated with Postpartum Depressive Symptoms

As shown in Table 4, multivariable logistic regression analysis was used to identify factors independently associated with postpartum depressive symptoms after controlling for potential confounders. Low sleep quality remained associated with higher odds of postpartum depressive symptoms (aOR = 5.95, 95% CI: 2.78–12.70, *p* < 0.001), whereas high perceived social support remained associated with lower odds of these symptoms (aOR = 0.33, 95% CI: 0.15–0.70, *p* = 0.004). Adherence to doing-the-month practices was not a statistically significant associated factor in the adjusted model.

In the supplementary adjusted analysis, living with a mother-in-law was associated with higher odds of postpartum depressive symptoms (aOR = 2.28, 95% CI: 1.07–4.88, *p* = 0.034; see Supplementary Table S2).

The multivariable logistic regression model demonstrated an acceptable fit, as indicated by the Hosmer–Lemeshow test (*p* = 0.654). The Cox and Snell R^2^ and Nagelkerke R^2^ values were 0.26 and 0.35, respectively. The model correctly classified 76.8% of participants (see Supplementary Table S3). The variance inflation factor values ranged from 1.05 to 1.24, indicating that multicollinearity was not a concern.

## 4. Discussion

This study found a relatively high prevalence of postpartum depressive symptoms among women attending postnatal check-ups at two hospitals in Yunnan Province. This prevalence was higher than the overall prevalence reported for China [3] but was comparable to that reported in a previous study conducted in Yunnan Province, Southwest China [4]. This relatively high prevalence may be partly explained by methodological differences. Prevalence estimates vary according to the cut-off score applied, with lower cut-off scores generally yielding higher rates of positive screening results. The EPDS cut-off score of ≥10 used in this study has been supported as an appropriate screening threshold among Chinese postpartum women [21]. In this study, the EPDS score distribution was centered near the selected threshold, suggesting that a number of participants had scores close to the cut-off score. Therefore, applying a higher threshold would likely have resulted in a lower prevalence estimate. Furthermore, depressive symptoms are frequently observed in the early postpartum period and tend to decline gradually over time [30]. Assessment within 6 weeks postpartum may partly explain the higher prevalence. From a biopsychosocial perspective, postpartum depressive symptoms may be associated with multiple interacting factors. Biologically, Yunnan’s high-altitude environment may increase the risk of poor sleep and anxiety [31]. Psychosocially, the transition to motherhood may contribute to postpartum depressive symptoms through difficulties in role adjustment, emotional distress, and changes in social support and interpersonal relationships [32]. Moreover, Yunnan is a mountainous and ethnically diverse province with numerous rural and ethnic minority communities, where access to maternal health care resources may be relatively limited [33]. Traditional cultural beliefs among ethnic minority women may influence their willingness to access maternal health services [34]. These biological and psychosocial conditions may contribute to greater susceptibility to postpartum depressive symptoms.

Low sleep quality was independently associated with postpartum depressive symptoms. This finding may be explained by the postpartum caregiving context. During the early postpartum period, maternal sleep commonly remains fragmented, and nighttime infant care/feeding contributes to poorer nocturnal sleep [35]. Because the participants were assessed at 6 weeks postpartum, the association between low sleep quality and depressive symptoms may have been particularly strong during this period. In addition, sleep disturbance may be associated with postpartum depressive symptoms through physiological stress-related mechanisms. For example, depression has been linked to dysregulation of the hypothalamic–pituitary–adrenal (HPA) axis, including increased HPA axis activity, altered glucocorticoid release, and impaired negative feedback regulation [10]. In the Yunnan context, high-altitude environments may further increase vulnerability to low sleep quality through hypoxia and a lower partial pressure of inspired oxygen [31]. This finding is consistent with that of a previous study conducted in China [22], which reported a positive association between poor sleep quality and postpartum depressive symptoms. However, given the cross-sectional design, temporal directionality cannot be established. Sleep disturbance and depressive symptoms were assessed simultaneously; therefore, sleep quality is interpreted as a concurrently associated risk indicator rather than as a confirmed antecedent cause. Nevertheless, prospective evidence from other populations supports the plausibility of postpartum sleep quality as a predictor of subsequent depressive symptoms [15,16], warranting further investigation using longitudinal studies in Yunnan.

Women with high perceived social support had lower odds of postpartum depressive symptoms than women with lower perceived social support. This finding may be partly explained by the stress-buffering function of perceived social support, which may strengthen coping resources and indirectly reduce postpartum stress by enhancing marital satisfaction and maternal postnatal attachment [36]. Importantly, perceived social support reflects an individual’s subjective appraisal of available support rather than the support actually received, and these constructs are conceptually distinct [37]. This distinction may be particularly relevant in Yunnan Province. In culturally diverse and multi-ethnic settings, postpartum women may rely more heavily on family members and close social networks, particularly when clinical practices are perceived to conflict with local beliefs, fears, or traditional customs, which may discourage them from using maternal health services [34]. However, the availability of support may not necessarily be perceived as adequate, particularly when family support is shaped by cultural expectations, intergenerational differences, and potential conflicts during the postpartum period [38]. This finding is consistent with that of a previous study conducted in China [11]. The association between perceived social support and postpartum depressive symptoms may be bidirectional: while low social support may contribute to depressive symptoms, depression may in turn lead to social withdrawal and reduced perceived support. Given the cross-sectional design, causal direction cannot be determined. Nonetheless, perceived social support remains a clinically accessible and modifiable factor, and its concurrent association with lower odds of PPD in this sample supports its inclusion in routine postpartum screening protocols.

In this study, no significant association was observed between adherence to doing-the-month practices and postpartum depressive symptoms. Doing-the-month encompasses multiple practices, such as dietary, hygiene, and activity restrictions, which may have differential associations with postpartum mental health [13]. Therefore, the use of an overall ADP score may have masked the associations of individual practices with postpartum depressive symptoms. In addition, cultural variation across ethnic groups in Yunnan may not have been fully captured by the ADP scale, as evidence from rural Yunnan suggests that preferences for postpartum health services and cultural beliefs may vary across ethnic groups and local contexts [18]. This finding is consistent with a recent review in which most of the included studies reported no substantial association between doing-the-month practices and PPD [19]. An additional consideration is that reverse causality may partly explain the null finding: women experiencing depressive symptoms may have reduced motivation and energy for confinement practices, meaning lower adherence could be a consequence rather than a cause of PPD. This interpretation is consistent with the cross-sectional nature of the data, in which temporal sequence cannot be established. Future longitudinal studies are needed to clarify whether doing-the-month practices influence PPD onset, or whether depressive symptoms precede disengagement from these practices.

An additional exploratory finding was that living with a mother-in-law was significantly associated with higher odds of postpartum depressive symptoms in the multivariable model. This finding may be partly attributed to the dual role of mothers-in-law within the Chinese postpartum care context. Mothers-in-law frequently participate in doing-the-month practices and infant care; however, interpersonal conflict may diminish the potential benefits of family support and contribute to greater emotional distress [38]. Nevertheless, as this was not a prespecified primary factor and family relationship quality was not assessed, the finding should be interpreted cautiously.

Overall, these findings support the use of a biopsychosocial perspective in understanding postpartum depressive symptoms among women in Yunnan Province. Postpartum screening and care should therefore include assessments of sleep quality, perceived social support, and family context.

### 4.1. Limitations

Several limitations should be considered when interpreting these findings. First, the cross-sectional design precludes causal inference; because all variables were assessed simultaneously, the temporal relationships among sleep quality, perceived social support, doing-the-month practices, and postpartum depressive symptoms cannot be determined. These factors are therefore interpreted as concurrently associated risk indicators rather than as established antecedents of PPD. Second, convenience sampling at two hospitals in Yunnan Province limits the generalizability of the findings, which should not be extrapolated to the broader postpartum population at the provincial or national level. A population-based or multisite probability sample would be required to generate representative estimates of provincial prevalence. Third, the use of self-report questionnaires may have introduced recall and social desirability biases. Fourth, dichotomizing continuous variables at the sample median may have resulted in information loss and reduced statistical power, and no sensitivity analysis using continuous scores was conducted. Fifth, the ADP scale has not been validated within the multi-ethnic context of Yunnan; therefore, its measurement accuracy may have been limited, potentially contributing to the non-significant finding for doing-the-month practices.

### 4.2. Implications for Clinical Practice and Future Research

These findings have practical implications for nursing practice and education and provide directions for future research in Yunnan Province, where postpartum women may have diverse ethnic, cultural, geographic, and family backgrounds. Nurses should enhance early screening for postpartum depressive symptoms during the first 6 weeks after childbirth and routinely assess sleep quality, perceived social support, and family living arrangements. In this multi-ethnic context, culturally sensitive care is essential because traditional postpartum practices, such as doing-the-month, and patterns of family support may shape women’s postpartum mental health experiences.

Nursing education should strengthen preparation in postpartum mental health screening and biopsychosocial assessment, with attention to sleep quality, social support, and culturally related postpartum practices. Future longitudinal and multi-site studies should clarify temporal relationships, recruit more representative samples, analyze scale scores continuously where appropriate, compare alternative EPDS cut-off scores, and evaluate specific components of doing-the-month practices across ethnic groups. Intervention studies are also needed to test culturally adapted strategies for reducing postpartum depressive symptoms.

## 5. Conclusions

This cross-sectional study found a high prevalence of postpartum depressive symptoms among women attending postnatal check-ups at two hospitals in Yunnan Province. Within this hospital-based sample, low sleep quality was associated with higher odds of postpartum depressive symptoms, whereas high perceived social support was associated with lower odds; adherence to doing-the-month practices was not significantly associated with postpartum depressive symptoms. Given the exploratory nature of these findings, they should not be generalized to the broader population of Yunnan Province without further replication using probability-based sampling. Nevertheless, the findings underscore the importance of early screening for postpartum depressive symptoms and the routine assessment of sleep quality, perceived social support, and family context during postpartum care. Furthermore, culturally sensitive nursing interventions should be developed to address the diverse needs of postpartum women within the multi-ethnic context of Yunnan Province.

## Figures and Tables

**Table 1:** Demographic Characteristics and Postpartum Depression Symptoms of the Participants (n = 168).

Demographic Characteristics	Frequency (n)	Percentage (%)
Maternal Age		
Age < 25	18	10.70
Age ≥ 25	150	89.30
Ethnicity		
Han	122	72.60
Non-Han	46	27.40
Education Level		
High School or Lower	27	16.10
College/University or Higher	141	83.90
Employment Status		
Unemployed	61	36.30
Employed	107	63.70
Parity		
Primiparous	107	63.70
Multiparous	61	36.30
Living with Mother-in-Law		
No	82	48.80
Yes	86	51.20
Postpartum Depression Symptoms		
NO (EPDS < 10)	93	55.40
Yes (EPDS ≥ 10)	75	44.60

**Table 2:** Descriptive Statistics of Study Variables (n = 168).

Variable	Median (IQR)	Mean ± SD	Min–Max
Postpartum Depression	9 (7)	9.83 ± 5.34	0–25
Sleep Quality	23 (9)	23.79 ± 6.09	12–42
Perceived Social Support	66 (19)	63.46 ± 14.23	12–84
Adherence to Doing-the-Month	52 (36)	50.50 ± 21.98	2–104

**Table 3:** Univariable Logistic Regression Analysis of Postpartum Depressive Symptoms (n = 168).

Factor	Crude OR	95% CI	*p*
Low Sleep Quality	6.71	3.34–13.46	<0.001
High Social Support	0.23	0.12–0.44	<0.001
High Adherence to Doing-the-Month	0.70	0.38–1.29	0.249

**Table 4:** Multivariable Logistic Regression Analysis of Factors Associated with Postpartum Depressive Symptoms (n = 168).

Factor	B	SE	aOR	95% CI	*p*
Low Sleep Quality	1.78	0.39	5.95	2.78–12.70	<0.001
High Social Support	−1.11	0.39	0.33	0.15–0.70	0.004
High Adherence to Doing-the-Month	−0.29	0.40	0.75	0.34–1.64	0.471

## Data Availability

The original contributions presented in the study are included in the article and Supplementary Materials; further inquiries can be directed to the corresponding author.

## Supplementary Materials

The following supporting information can be downloaded at: https://www.mdpi.com/article/10.3390/nursrep16070215/s1, Table S1. Univariable analysis of demographic factors; Table S2. Multivariable analysis of demographic factors; Table S3. Model fit statistics.

## Public Involvement Statement

No public involvement in any aspect of this research.

## Guidelines and Standards Statement

The reporting of this study followed the Strengthening the Reporting of Observational Studies in Epidemiology (STROBE) guidelines for observational cross-sectional research.
