# Supplementary material for: Prevalence and Factors Associated with Postpartum Depressive Symptoms Among Women Attending Postnatal Check-Ups at Two Hospitals in Yunnan Province, China: A Cross-Sectional Study
Source: Nurs Rep. 2026 Jun 25;16(7):215. doi: 10.3390/nursrep16070215 (PMC13415229; doi:10.3390/nursrep16070215)
Supplement: Supplementary file 1 [file nursrep-16-00215-s001.zip › nursrep-4352639-supplementary.pdf]

Supplementary Table

As shown in Supplementary Table S1, none of the demographic variables were significantly associated with postpartum depression in the univariable analysis. Maternal age, ethnicity, education level, employment status, parity, and living with mother-in-law were not statistically significant predictors of postpartum depression.

Supplementary Table S1. Univariable Associations Between Demographic Variables and Postpartum Depression (n = 168)

Predictor	Crude OR	95% CI	p
Age ≥25	0.61	0.23–1.64	.328
Non-Han	0.83	0.42–1.65	.593
College/University or Higher	0.85	0.37–1.93	.689
Employed	0.55	0.29–1.04	.064
Multiparous	0.71	0.38–1.35	.298
Living with mother-in-law	1.56	0.85–2.88	.153

As shown in Supplementary Table S2, after adjustment, only living with mother-in-law remained significantly associated with postpartum depression. Women living with mother-in-law had higher odds of postpartum depression than those not living with mother-in-law (aOR = 2.28, 95% CI: 1.07–4.88, $p = .034$). The other demographic variables were not statistically significant in the multivariable model.

Supplementary Table S2. Multivariable Associations Between Demographic Variables and Postpartum Depression (n = 168)

Predictor	B	SE	aOR	95% CI	p
Age ≥25	−0.17	0.67	0.84	0.23–3.12	.798
Non-Han	−0.45	0.43	0.64	0.27–1.49	.298
College/University or Higher	0.08	0.55	1.09	0.37–3.16	.881
Employed	−0.29	0.43	0.75	0.32–1.75	.507
Multiparous	−0.52	0.40	0.60	0.27–1.30	.192
Living with mother-in-law	0.82	0.39	2.28	1.07–4.88	.034

Model fit statistics for the final logistic regression model are presented in Supplementary Table S3. The model demonstrated acceptable goodness of fit, as indicated by a non-significant Hosmer–Lemeshow test ($p = .654$). The Cox & Snell R^2 was 0.26 and the Nagelkerke R^2 was 0.35, suggesting that approximately 26%–35% of the variance in postpartum depression could be explained by the predictors included in the model. The overall classification accuracy of the model was 76.8%.

Supplementary Table S3. Model Fit Statistics for the Multivariable Logistic Regression Model

Statistic	Value
−2 Log likelihood	179.90
Cox & Snell R^2	0.26
Nagelkerke R^2	0.35
Hosmer–Lemeshow p	.654
Overall accuracy	76.8%

Note: $p > .05$ indicates adequate fit.
